# Convulsive seizures from experimental focal cortical dysplasia occur independently of cell misplacement

**DOI:** 10.1038/ncomms11753

**Published:** 2016-06-01

**Authors:** Lawrence S. Hsieh, John H. Wen, Kumiko Claycomb, Yuegao Huang, Felicia A. Harrsch, Janice R. Naegele, Fahmeed Hyder, Gordon F. Buchanan, Angelique Bordey

**Affiliations:** 1 Departments of Neurosurgery, and Cellular and Molecular Physiology, Yale University School of Medicine, New Haven, Connecticut 06520-8082, USA; 2 Department of Neurology, Yale University School of Medicine, New Haven, Connecticut 06520-8082, USA; 3 Magnetic Resonance Research Center, Yale University School of Medicine, New Haven, Connecticut 06520-8082, USA; 4 Department of Biology, Wesleyan University, Middletown, Connecticut 06459, USA

## Abstract

Focal cortical dysplasia (FCD), a local malformation of cortical development, is the most common cause of pharmacoresistant epilepsy associated with life-long neurocognitive impairments. It remains unclear whether neuronal misplacement is required for seizure activity. Here we show that dyslamination and white matter heterotopia are not necessary for seizure generation in a murine model of type II FCDs. These experimental FCDs generated by increasing mTOR activity in layer 2/3 neurons of the medial prefrontal cortex are associated with tonic-clonic seizures and a normal survival rate. Preventing all FCD-related defects, including neuronal misplacement and dysmorphogenesis, with rapamycin treatments from birth eliminates seizures, but seizures recur after rapamycin withdrawal. In addition, bypassing neuronal misplacement and heterotopia using inducible vectors do not prevent seizure occurrence. Collectively, data obtained using our new experimental FCD-associated epilepsy suggest that life-long treatment to reduce neuronal dysmorphogenesis is required to suppress seizures in individuals with FCD.

## Abstract (web summary)

The etiology of focal cortical dysplasia (FCD) is not fully understood. Here authors generate an mTORC1 overactivation mouse model that recapitulates hallmarks of type II FCDs, including spontaneous seizures, and suggest that neuronal defects, rather than macrostructural changes, lead to seizures.

## Graphical abstract

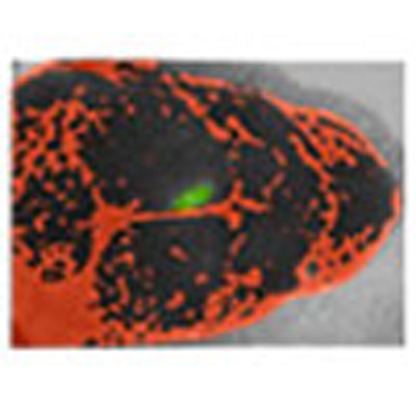

Malformations of cortical development (MCDs) are often (80–90%) associated with epilepsy and developmental delay in young children. They result from abnormalities in cortical development and include several types of cortical dysplasia that are classified based on several parameters, including the developmental stage when the defect occurs, the underlying genetic mutations and histopathology^1^,^2^,^3^,^4^,^5^. Focal MCDs are commonly detected in neurodevelopmental disorders such as focal cortical dysplasia (FCD, also referring to the malformation itself) and tuberous sclerosis complex (TSC), and are the major cause of medically refractory epilepsy^3^,^4^,^6^. The only therapeutic option is surgical resection, but only 30–50% of these patients will properly manage their seizures following surgery^7^,^8^,^9^,^10^,^11^. There is a clear need to improve our understanding of the aetiology of these focal MCDs and the mechanisms of epileptogenesis to identify novel treatments. However, progress towards such understanding and developing effective treatments have remained challenging without an experimental model of FCD that recapitulates the characteristics of human epilepsy-associated FCDs.

Previous studies have reported several experimental murine models of MCDs with a particular emphasis on TSC and the associated focal MCDs (called cortical tubers) because the genetics of TSC was known before that of FCDs^12^,^13^,^14^,^15^ (see refs 16, 17, 18 for earlier and additional references). In TSC, *TSC1* or *TSC2* are mutated in individuals leading to upregulated mechanistic target of rapamycin complex 1 (mTORC1) activity as observed in type II and III FCDs^19^,^20^,^21^. Most models have been generated in conditional transgenic mice crossed with different driver lines that express Cre recombinase (Cre) under the control of cell-type-specific promoters. Following selective mTORC1 upregulation in glutamatergic neurons and astrocytes or in developing neurons, affected mice display severely malformed forebrains, seizures and premature death. Although these models provide information about the identity of the affected cell types leading to specific defects seen in MCDs and are valuable to test the efficacy of the mTORC1 blocker rapamycin on preventing seizure activity, there are several limitations that preclude their use for mechanistic studies of epileptogenesis. These include the presence of widespread forebrain alterations instead of focal malformations surrounded by normal brain tissue, premature death of the animals, difficulties in assessing whether molecular changes result from or contribute to seizure activity and difficulties in performing genetic rescue. To address these issues, several groups have generated focal lesions using *in utero* electroporation to manipulate components of the mTORC1 signalling pathway at a given time during development^22^,^23^,^24^. However, in prior studies the focal lesions did not lead to the occurrence of spontaneous seizures.

We therefore set out to develop a model of FCD-associated spontaneous, recurrent convulsive seizures. Using *in utero* electroporation to increase mTORC1 activity in specific neuronal populations in the developing cortex, we report the generation of focal malformations that display the hallmarks of type II FCDs, that is, cortical dyslamination, white matter heterotopia and neuronal dysmorphogenesis. Additional FCD characteristics include focal cortical enlargement and alterations in connectivity detected using non-functional magnetic resonance imaging (MRI). Importantly, FCDs generated in the medial prefrontal cortex (mPFC) lead to recurrent tonic–clonic seizures and gliosis that are absent when FCDs are generated in the somatosensory cortex (SSC). These experimental FCDs can thus be instrumental to identify and differentiate causal alterations from epiphenomenon associated with seizures. Finally, we used this newly developed FCD model to examine the efficiency of rapamycin at rescuing cortical defects and associated seizures and to address whether preventing misplacement and heterotopia would be sufficient to prevent seizure generation. We found that rapamycin treatments initiated after birth prevent all structural and cellular defects as well as seizure activity, suggesting that embryonic treatments are not required to rescue FCDs and eliminate seizures. To address the second issue, we used two approaches: withdrawal of rapamycin and design of genetic approaches to induce neuronal dysmorphogenesis in layer 2/3 of the mPFC without macrostructural abnormalities. Resulting data suggest that neuronal dysmorphogenesis in a subpopulation of layer 2/3 mPFC neurons is sufficient to generate recurrent, convulsive seizures. This finding implies that a life-long treatment to persistently reduce neuronal dysmorphogenesis is required to reduce or eliminate seizure activity.

## Results

### Generation of FCDs in the mPFC leads to spontaneous seizures

**Figure 1:**
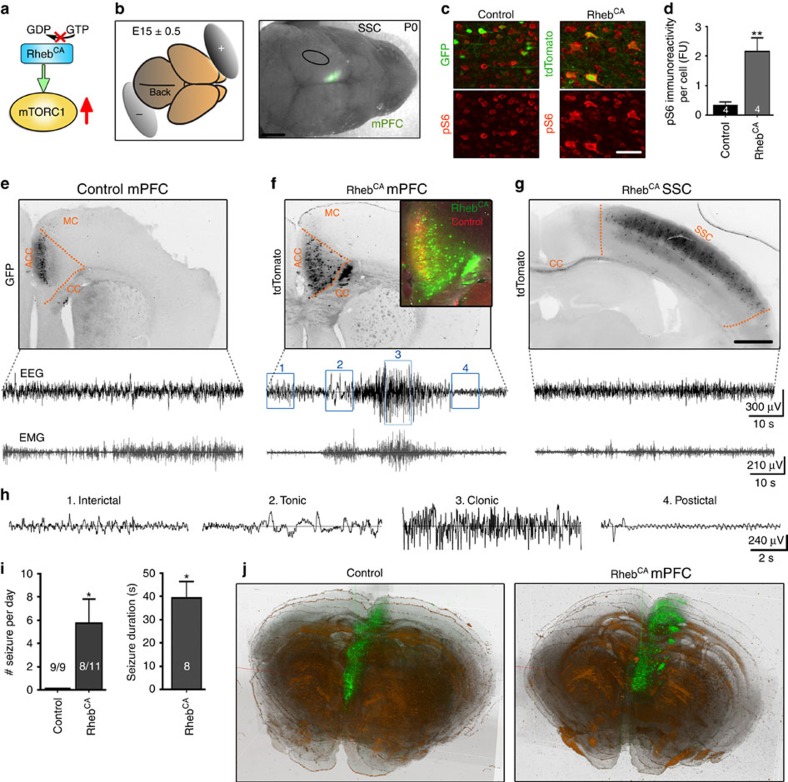
Generation of experimental FCD-associated convulsive seizures. (**a**) Simplified diagram of the constitutively active Rheb (Rheb^CA^) upstream of mTORC1. (**b**) Diagram of the electrode placement around an E15±0.5 fetal brain (left). Photograph of a P0 mouse head with GFP fluorescence in the medial prefrontal cortex (mPFC) following electroporation at E15 (right). The ellipse highlights the location of the somatosensory cortex (SSC). (**c**) Confocal images of pS6 immunostaining (red) and GFP and tdTomato fluorescence (green) in coronal sections from 2-month-old mice containing neurons electroporated with either Rheb^CA^+tdTomato^CAG^ or GFP^CAG^ alone in the mPFC. Scale bar, 40 μm. (**d**) Bar graphs of total pS6 immunoreactivity per cell (FU: arbitrary fluorescence unit, *N*=4 per condition) from data shown in (**c**). **P<0.01 (Student's *t*-test). (**e**–**g**) Images of coronal sections (top) containing neurons electroporated with control plasmid in the mPFC (**e**), with Rheb^CA^ in the mPFC (**f**) or in the SSC (**g**), and corresponding representative examples of EEG (black) and EMG (grey) traces (bottom). Scale bar, 1 mm. (**h**) Expanded time window (10 s) view of events found in the EEG trace shown in **f**, numbered correspondingly to the blue boxes. (**i**) Bar graphs show the number (#) of seizures/day and seizure duration in animals with seizures (*N*=8) from the mPFC Rheb^CA^-expressing cohort. Control animals (*N*=9) electroporated with a control plasmid did not exhibit any seizures. *P<0.05 (Student's *t*-test). (**j**) Three-dimensional reconstruction of mouse brains containing neurons electroporated with either Rheb^CA^+tdTomato^CAG^ or a control plasmid GFP^CAG^, both pseudocolored green to ease visualization. ACC, anterior cingulate cortex; CC, corpus callosum; MC, motor cortex. Error bars, s.e.m.

The mTOR complex 1 (mTORC1) signalling pathway is a shared causal molecular link between type II FCD and TSC as well as other MCD-related disorders including ganglioglioma^25^, hemimegalencephaly^14^,^26^ and STRAD alpha deficiency^27^. In FCD and TSC, focal MCDs are generated during embryonic life by an increase in mTORC1 activity in a subset of developing cells^28^. To recapitulate this molecular event, we used *in utero* electroporation to express a plasmid encoding a constitutively active form of Rheb (Rheb^CA^), the canonical activator of mTORC1 (Fig. 1a). This approach allowed us to target a subset of neural progenitor cells at a specific embryonic day (E) and in a specific cortical region (Fig. 1b). We preferentially targeted neurons destined for the mPFC because 50–90% of the focal malformations are in the frontal lobes of patients^29^,^30^,^31^,^32^. Moreover, frontal lobe malformations are often associated with seizure activity and intractable epilepsy^31^,^32^. Electroporation at E15 (±0.5) selectively introduces plasmids into neural progenitor cells generating layer 2/3 cortical pyramidal neurons^33^. Most of the data are from 2- to 4-month-old animals, unless specified otherwise.

To examine whether Rheb^CA^ effectively increased mTORC1 activity, immunostaining for phosphorylated ribosomal protein S6 (pS6) was performed in coronal sections from 2-month-old mice electroporated with Rheb^CA^ and tdTomato-encoding plasmids (tdTomato^CAG^) or a green fluorescence protein-encoding control plasmid (GFP^CAG^). Phosphorylated S6 is commonly used as a marker of mTORC1 activity because mTORC1 activates p70 S6 kinase 1, which phosphorylates S6 (ref. 34). Rheb^CA^-expressing neurons displayed a significant increase in pS6 immunoreactivity compared with control neurons in 2-month-old mice, suggesting that the Rheb^CA^ vector efficiently increased mTORC1 activity (Fig. 1c,d). As expected, electroporation of control plasmids resulted in GFP labelling of cortical neurons positioned in layer 2/3 (Fig. 1e). By contrast, electroporation of Rheb^CA^ in littermates resulted in a scattering of labelled cortical neurons throughout all cortical layers as well as the corpus callosum (Fig. 1f). The striking difference in the placement of labelled neurons under control versus Rheb^CA^ conditions is further illustrated in the overlay in Fig. 1f. Importantly, 8 out of 11 mice expressing Rheb^CA^ in the mPFC displayed recurrent tonic-clonic seizures (Fig. 1f) while none of the mice expressing a control plasmid (*N*=9) displayed seizures (*P*<0.001, two-proportion *z*-test, Fig. 1e). Seizures were detected using epidural electroencephalography (EEG) and electromyography (EMG) recordings combined with video monitoring, starting at 2 months of age. EEG traces corresponding to the interictal, tonic, clonic and postictal periods of a seizure in mice expressing Rheb^CA^ in the mPFC are displayed in Fig. 1h in an expanded time scale. Power spectra of the inter- and post-ictal periods displayed peak frequencies of 2–3 and 4–5 Hz, which are consistent with resting-state activity (delta rhythm) and theta rhythm, respectively, as reported in human EEG recordings (Supplementary Fig. 1 and Video 1). All animals experiencing seizures displayed Racine stage 3–5 seizures following unilateral mPFC electroporation of Rheb^CA^ (*N*=8/11). On the basis of behavioural observations, we found that mice exhibited Racine stage 3–5 seizures as early as P19. Convulsive seizure activity did not result from electroporation of Rheb^CA^ in the SSC(N3, Fig. 1g), suggesting that the location of cortical malformation is a critical determinant in the development of epilepsy in these mice. These results also suggest that the mPFC is an epileptogenic area. Seizures occurred multiple times during the 3 days of consecutive monitoring with a mean rate of ∼6 (5.7±2.1, *N*=8) seizures per day and a mean duration of ∼40 s (39.4±7.0 s, *N*=8, Fig. 1i). Despite experiencing seizures, Rheb^CA^-electroporated mice displayed normal survival, as we had no deaths before performing the EEG recordings at 2–4 months of age. The extent of electroporation was routinely examined in all electroporated animals as illustrated in a three-dimensional reconstruction of the electroporated region in control and Rheb^CA^ condition (Fig. 1j). The core electroporated mPFC regions required for obtaining an epileptic phenotype spanned from pre-orbital cortex to the end of the cingulate cortex.

### mTOR-induced cortical dysplasia are type II FCDs

FCDs are divided into three groups based on the pathological alterations^35^. Type I FCDs display macrostructural abnormalities characterized by neuronal misplacement resulting in cortical dyslamination and the presence of white matter heterotopia. Type IIa FCDs contain cytomegalic neurons with altered morphology, in addition to the macrostructural abnormalities of Type I FCDs, and type IIb also include Balloon cells with a mixed neuro-glial phenotype. Finally, type III FCDs display additional lesions (for example, tumours or hippocampal sclerosis compared with type II FCDs).

**Figure 2:**
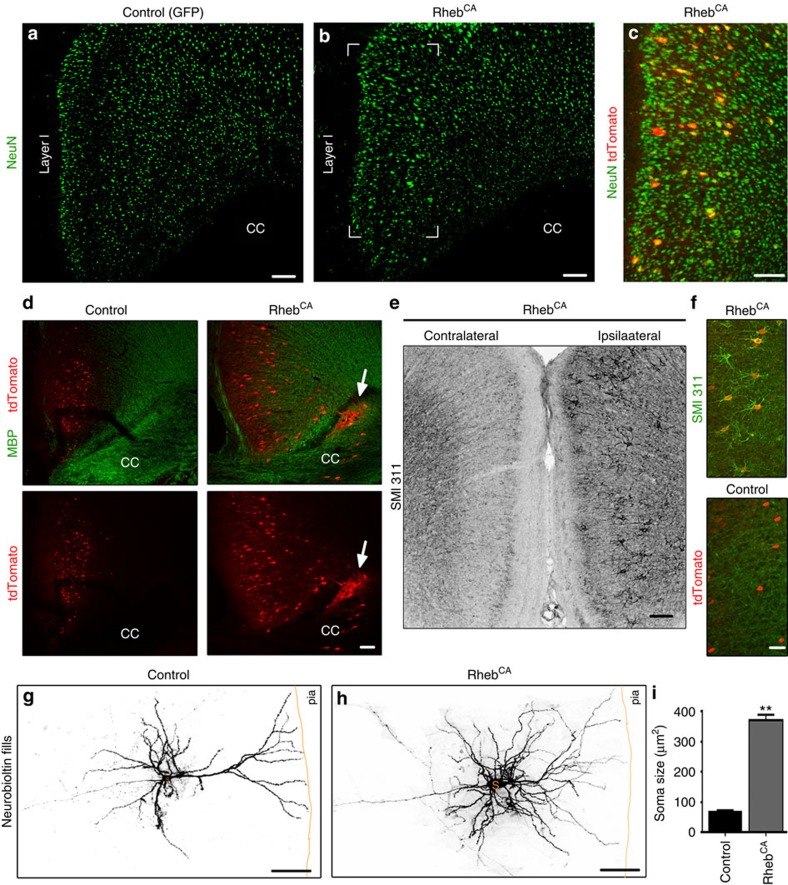
Experimental cortical malformations display typical features of type II FCDs. (**a**,**b**) NeuN immunostaining in coronal sections containing control (**a**) or Rheb^CA^ (**b**) expressing neurons. Electroporated neurons are not shown. Scale: 100 μm. (**c**) NeuN immunostaining (green) of tdTomato^+^ cells (red) in the Rheb^CA^ condition from the white rectangle shown in (**b**). Scale bar, 100 μm. (**d**) Myelin basic protein (MBP) immunostaining (green) of tdTomato^+^ cells (red) in the Rheb^CA^ condition illustrating myelin displacement and white matter heterotopia in the corpus callosum (CC). Scale bar, 100 μm. (**e**) SMI 311 immunostaining in a coronal section containing ipsilateral (Rheb^CA^ electroporated) and contralateral ACC. Electroporated neurons are not shown, but strong somatodendritic SMI 311 immunoreactivity is visible in ipsilateral neurons. Scale bar, 100 μm. (**f**) SMI 311 immunostaining (green) of tdTomato^+^ cells (red) in the Rheb^CA^ (from **e**) and control conditions. Scale bar, 50 μm. (**g**,**h**) Confocal images of control and Rheb^CA^-expressing neurons in layer 2/3 of the ACC. Neurons were microinjected with neurobiotin and post-stained with Alexa Fluor 633 conjugated Streptavidin for visualization. Scale bar, 100 μm. (**i**) Bar graphs of soma size of control and Rheb^CA^-expressing neurons in layer 2/3. **P<0.01 (Student's *t*-test). Error bars, s.e.m.

Consistent with neuronal misplacement as illustrated in Fig. 1, Rheb^CA^ electroporation at E15 led to cortical dyslamination detected with staining for a neuronal marker, NeuN (Fig. 2a,b). Electroporated cells (tdTomato^+^) were all NeuN positive, identifying them as neurons (Fig. 2c). The presence of neurons trapped in the white matter, that is, white matter heterotopia, led to myelin distortion in the corpus callosum, as shown by myelin basic protein immunoreactivity in coronal sections containing Rheb^CA^-expressing neurons (Fig. 2d). In addition to these macrostructural defects, Rheb^CA^-expressing neurons displayed abnormal somatic accumulation of neurofilaments (NF), as indicated by somatodendritic immunoreactivity to a pan NF-specific antibody SMI 311 (Fig. 2e,f), identical to dysmorphic neurons found in human FCDs^36^. NF light chain was subsequently identified as the filamentous form accumulating in the soma, which has not been previously reported (Supplementary Fig. 2). When Rheb^CA^-expressing neurons were filled with neurobiotin using whole-cell patch clamp recordings, we observed that they were cytomegalic with significantly increased soma sizes and dysmorphic dendritic trees compared with control (Fig. 2g–i). However, Rheb^CA^-expressing cells did not stain for markers commonly found in Balloon cells, that is, nestin or vimentin (Supplementary Fig. 3) (ref. 37).

Collectively, these data show that the experimental cortical malformations generated by E15 Rheb^CA^ electroporation display characteristics of type II FCDs.

### Gliosis and altered connectivity in FCDs

**Figure 3:**
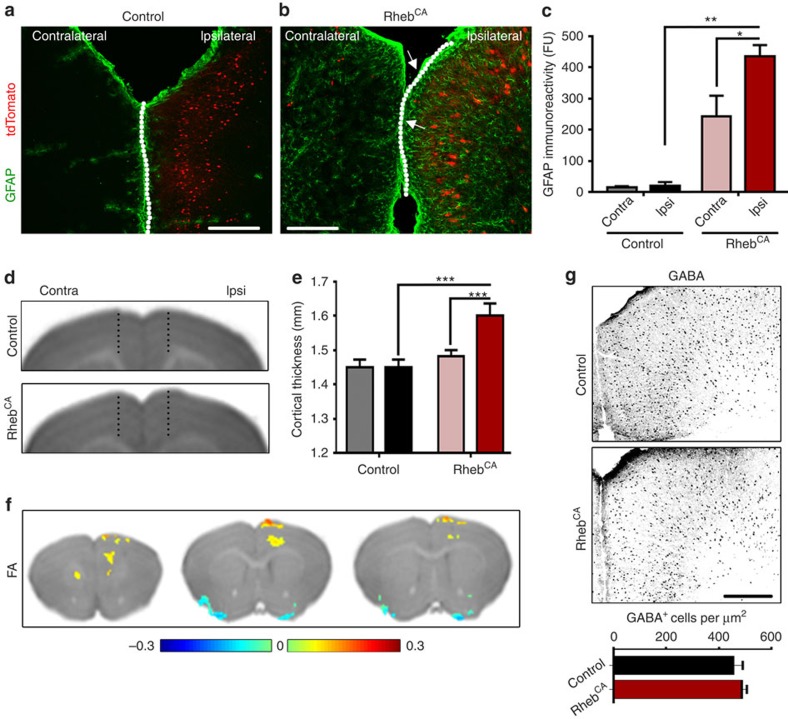
Gliosis but no change in GABAergic neuron density are visible in FCDs. (**a**,**b**) GFAP immunostaining (green) in coronal sections containing electroporated neurons (red) under control (**a**) or Rheb^CA^ condition (**b**). The dotted line and white arrows point to a bulging of the ACC containing Rheb^CA^-expressing cytomegalic neurons. Scale bar, 150 μm. (**c**) Bar graphs of fluorescence (Fu) intensity of GFAP immunoreactivity in both the ipsilateral (ipsi) and contralateral (contra) cortex under conditions shown in (**a**,**b**). *P*<0.01 (two-way analysis of variance (ANOVA)); *P<0.05, **P<0.01 (Sidak's multiple comparisons post-test). (**d**) T1-weighted MR images of control (**d**) and Rheb^CA^ (**e**) brains showing measuring bars for cortical thickness. (**e**) Quantification of cortical thickness shown in **d**,**e** with *N*=6 per condition. *P*<0.001 (two-way ANOVA); ***P<0.001 (Sidak's multiple comparisons post-test). (**f**) Serial tomograms from diffusion tensor imaging (DTI) with statistically significant (*P*<0.01) differences in fractional anisotropy (FA; Rheb^CA^ minus control, expressed in heat maps with scale at the bottom) between control (*N*=4) and Rheb^CA^ (*N*=4) transfected brains, overlaid on top of anatomical scans of a control brain. (**g**) Top, GABA immunostaining in coronal sections from mice electroporated with control or Rheb^CA^ plasmid. Scale bar, 300 μm. Bottom, bar graph of the density of GABAergic cells in control and Rheb^CA^-expressing sections. Error bars, s.e.m.

FCDs associated with epilepsy in humans are accompanied by astrogliosis^38^. We thus immunostained electroporated brains for glial fibrillary acidic protein (GFAP), a classical marker of astrogliosis. We detected significant astrogliosis in FCD-associated with epilepsy (Fig. 3a–c). The astroglial reactivity occurred in and around the FCDs as well as throughout the entire cortex including the contralateral side, suggesting that generalizing seizure activity triggers gliosis (Fig. 3a–c). There was a significantly greater increase in GFAP immunoreactivity in and surrounding the FCDs compared with the contralateral cortex devoid of FCDs. One possible interpretation is that the presence of abnormal neurons with increased mTORC1 activity somehow sensitizes surrounding astrocytes to react to epileptiform activity, consistent with the absence of gliosis in FCDs without seizures (Supplementary Fig. 4). These data are in agreement with a previous report that used *in utero* electroporation in conditional *Tsc1* knockout mice^22^.

To further examine the histological characteristics of experimental FCDs, we measured the thickness of the cortical mantle, as prior work has reported increased mantle thickness in human FCDs^39^. A marked increase was observed, particularly at the midline between the ipsilateral, electroporated and the contralateral ACCs (Fig. 3a–b, white arrows and dotted line). To better quantify cortical thickness without tissue distortion from mounting on slides, we obtained T1-weighted MR images from 3- to 4-month-old control and Rheb^CA^-electroporated animals (Fig. 3d). Cortical thickness was measured from the horn of the corpus callosum to the top of cortex. Cortical thickness ipsilateral to the FCD was significantly greater than both the contralateral side and control brains (Fig. 3d,e).

Structural abnormalities in connectivity due to either cell dysmorphogenesis or heterotopia can be detected with diffusion tensor imaging (DTI) in both animals and humans^40^,^41^,^42^. In particular, DTI has been a powerful approach for measuring alterations in white matter of individuals with FCDs; the measurement is reported as fractional anisotropy (FA) and is based on the omnidirectional diffusion of water molecules in axon tracts and fibres, an indication of macroscopic tissue properties. We thus performed *ex vivo* DTI and observed a statistically significant increase in FA in the cortical region containing the experimental FCD (Fig. 3f). This was particularly true for the deep cortical layers suggesting severe microstructural abnormalities consistent with the presence of cytomegalic and ectopic neurons in deep cortical layers. DTI images were highly consistent across animals and could thus be used as a diagnostic tool as well as non-invasive measurements of FCD volume for a longitudinal drug study.

Considering the misplacement of cytomegalic pyramidal neurons, we next explored whether GABAergic interneurons, known to migrate during embryonic periods of development from the ganglionic eminence into the developing cortex, would also be misplaced. We thus performed immunostaining for GABA, which is expected to label all types of GABAergic interneurons. Contrary to our expectation, there was no difference in the density of GABAergic interneurons in the cortical region containing FCD compared with control (Fig. 3g). While these data imply that FCDs do not decrease the number of GABAergic interneurons, further studies would need to explore the functionality of GABAergic connections in these malformations.

### Postnatal rapamycin treatment rescues FCDs and seizures

**Figure 4:**
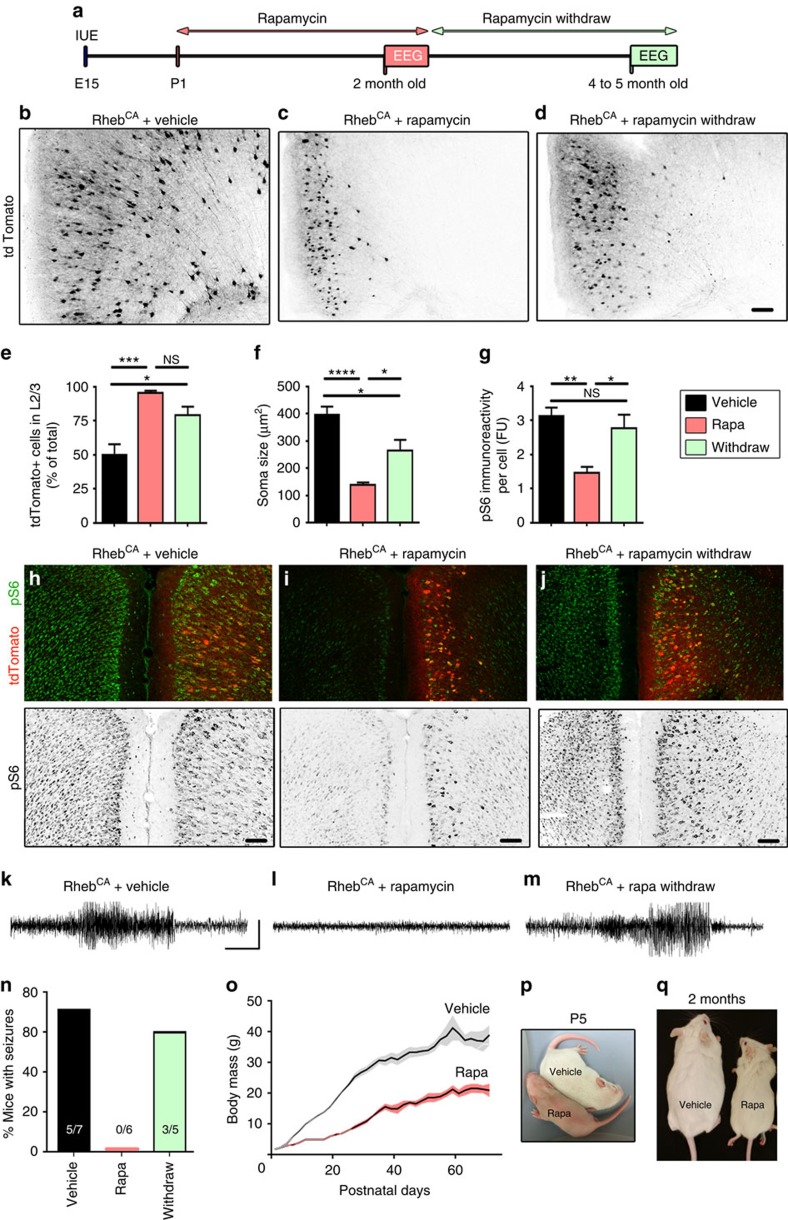
FCDs and seizures are prevented by chronic postnatal rapamycin treatments but return following rapamycin withdrawal. (**a**) Diagram illustrating the experimental protocol, including rapamycin treatment at 1 mg kg^−1^ every 48 h. (**b**–**d**) B&W images of Rheb^CA^-electroporated neurons expressing tdTomato in coronal sections from mice treated with either vehicle (**b**) or rapamycin (**c**), or following rapamycin withdrawal (**d**). Scale bar: 100 μm. (**e**) Bar graphs of the percentage of tdTomato^+^ Rheb^CA^ neurons in layer 2/3 in mice treated with vehicle (*N*=7) or rapamycin (*N*=6), or following rapamycin withdrawal (*N*=5). *P*<0.0007, one-way analysis of variance (ANOVA) followed by Tukey *post hoc*, *P<0.05; ***P<0.001, ****P<0.0001. (**f**,**g**) Bar graphs of tdTomato^+^ Rheb^CA^ neuron soma size (**f**) and pS6 immunoreactivity per cell (**g**) in mice treated with vehicle (*N*=7) or rapamycin (Rapa, *N*=6), or following rapamycin withdrawal (*N*=5). *P*<0.0001 and *P*<0.0026, respectively, one-way ANOVA followed by Tukey post hoc. (**h**–**j**) Images of Rheb^CA^-electroporated neurons expressing tdTomato and co-stained for pS6 (green and B&W for pS6 only) in coronal sections from mice treated with either vehicle (**h**) or rapamycin (**i**), or following rapamycin withdrawal (**j**). Scale bar, 100 μm. (**k**–**m**) Representative examples of EEG recordings in mice containing Rheb^CA^-electroporated cells treated with either vehicle (**k**) or rapamycin (**l**), or following rapamycin withdrawal (**m**). Scale bars, 14 s, 500 μV. (**n**) Bar graphs of the percentage of Rheb^CA^-electroporated mice displaying seizure activity that were treated with vehicle (black) or rapamycin (red), or following rapamycin withdrawal (green). (**o**) Plot of the body mass as a function of postnatal days of the animals treated with vehicle (*N*=7) or rapamycin (*N*=6). Mean (solid black line)±s.e.m. (colour shaded areas). *P*<0.0001 (two-way ANOVA with Sidak's multiple comparisons post-test). Significance starts at P13 of age. (**p**,**q**) Images of littermates at P5 (**p**) and 2 months (**q**) of age treated with vehicle or rapamycin. Error bars, s.e.m.

Rapamycin has been tested in several animal models of mTORC1-dependent malformation-induced seizures and has different rescue effects on brain alterations, seizure activity and premature death depending on the treatment regimens^13^,^43^,^44^,^45^,^46^,^47^,^48^. However, quantification of cellular changes with rapamycin has been difficult to assess considering that the whole forebrain is markedly affected in those earlier studies. To test the efficacy of a chronic regimen of postpartum therapy, rapamycin was injected intraperitoneally at 1 mg kg^−1^ from postnatal day (P) 1 to 2 months of age every 48 h (Fig. 4a). This treatment fully rescued cortical defects, compared with saline-injected animals (Fig. 4b,c,e–i). In the rapamycin-treated group, most neurons reached their proper location in layer 2/3 (Fig, 4c-e) and white matter heterotopia were absent (Fig. 4c). In addition, soma size and pS6 immunoreactivity were normalized following rapamycin treatment, compared with vehicle-treated animals (Fig. 4f–i). Consistent with the structural rescue, animals treated with rapamycin did not exhibit any convulsive seizures (*N*=6), while five of seven vehicle-treated animals displayed spontaneous, recurrent convulsive seizures (*N*=7, *P*<0.01, two-proportioned *z*-test, Fig. 4k,l,n). Despite a full rescue of neuronal defects and convulsive behaviour, rapamycin significantly delayed postnatal development, as indicated by three developmental milestones, that is, body mass index (Fig. 4o–q), fur (coat) growth (Fig. 4p) and eye opening.

These data suggest that rapamycin treatment starting after birth is sufficient to rescue FCD and associated seizures. Our experimental FCD model is thus amenable to testing and quantifying the efficacy of drug treatments.

### Seizures recur post-rapamycin withdrawal

The most readily observable defects of FCDs are dyslamination due to neuronal misplacement and white matter heterotopia. We thus examined whether transient rapamycin treatment after birth prevented seizure activity, in addition to the macrostructural defects reported above. Rheb^CA^-electroporated mice were treated with rapamycin as mentioned above (intraperitoneally at 1 mg kg^−1^, every 48 h) from P1 to 2 months of age and then kept rapamycin free for 2–3 months, before performing EEG recordings (Fig. 4a). At 2- to 3-month post-rapamycin treatment, neurons were properly positioned in layer 2/3 and as such their placement was not significantly different from ongoing rapamycin-treated mice (Fig. 4d,e). In addition, soma size and pS6 immunoreactivity were significantly increased compared with vehicle-treated mice (Fig. 4f,g,j), suggesting that on rapamycin withdrawal mTORC1 activity increases and leads to expected morphological defects despite the fact that Rheb^CA^-expressing neurons were properly positioned in the appropriate cortical layers. Finally, following rapamycin withdrawal spontaneous seizures occurred in 3/5 mice (Fig. 4m,n), a ratio that is not significantly different from vehicle-treated mice (two-proportioned *z*-test).

These data suggest that transient rapamycin treatment is not sufficient to maintain a seizure-free state in Rheb^CA^-electroporated mice, and thus life-long rapamycin treatment would be required for individuals with FCDs. Importantly, our data also suggest that gross neuronal misplacement and heterotopia are not required for seizure activity in this FCD model. However, rapamycin treatment during a critical period of development may have permanent effects on other not readily visible developmental features. We thus designed another set of experiment to address whether macrostructural defects were required for the generation of seizures using a genetic approach.

### Seizures occur without dyslamination and heterotopia

**Figure 5:**
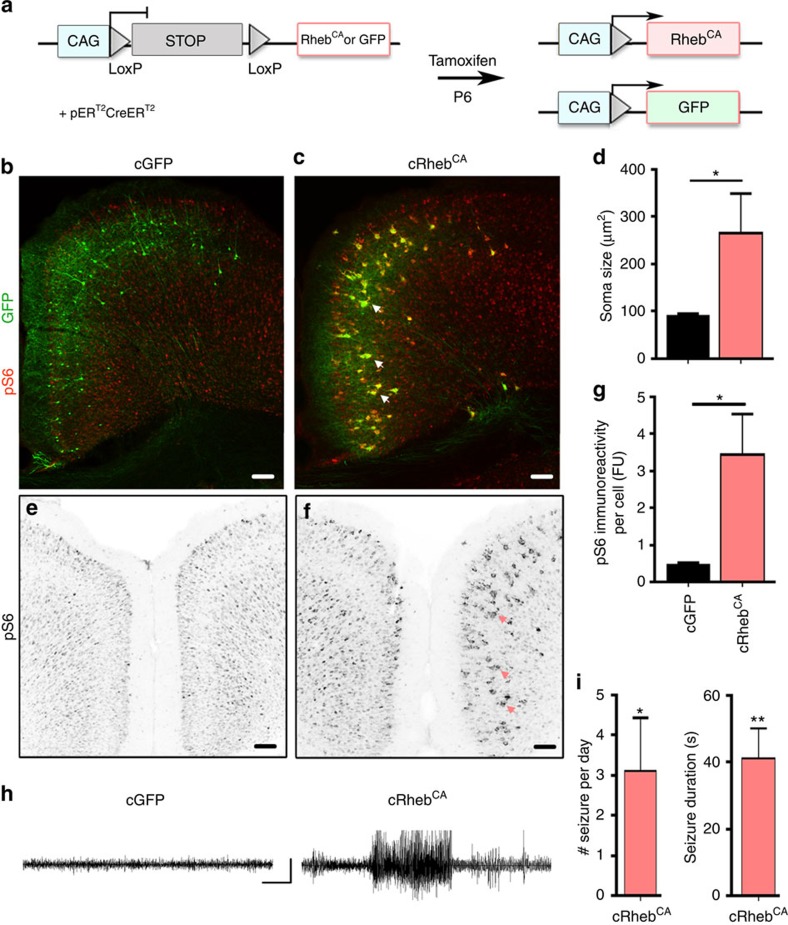
Dysmorphogenesis and cytomegaly of layer 2/3 mPFC neurons are sufficient to induce convulsive seizures. (**a**) Diagram illustrating the vectors and strategy used. (**b**,**c**,**e**,**f**) Images of cGFP+tdTomato^CAG^ or cRheb^CA^+GFP^CAG^-electroporated neurons co-stained for pS6 (red and B&W for pS6 only, (**e**,**f**)). Scale: 100 μm. Neurons were co-electroporated with an inducible Cre plasmid and injected with tamoxifen at P6. (**d**,**g**) Bar graphs of the soma size of cGFP^+^ (*N*=4) neurons and cRheb^CA+^ (*N*=3) neurons (**d**) and pS6 immunoreactivity per cell (**g**). *P<0.05 (Student's *t*-test). (**h**) Representative examples of EEG recordings from mice electroporated with plasmid encoding cGFP (left) and cRheb^CA^ (right). Scale bar, 10 s, 210 μV. (**i**) Bar graphs show the number (#) of seizures/day and seizure duration in seizing cRheb^CA^ animals (*N*=5). When compared to control (cGFP) littermates (*N*=6; *P<0.05; **P<0.01; Student's *t*-test). Error bars, s.e.m.

We generated a conditional Rheb^CA^ vector expressing LoxP sites containing a Stop cassette before Rheb^CA^ (cRheb^CA^) that leads to Rheb^CA^ expression on Cre expression (Fig. 5a diagram). Following electroporation of cRheb^CA^, GFP^CAG^ and a conditional Cre vector (cCre) at E15, tamoxifen was injected twice at P7 when most neurons have finished their migration. Control mice received cGFP-, cCre- and tdTomato^CAG^-encoding plasmids and identical tamoxifen injections. The cGFP-encoding plasmid allows for direct visualization of successful recombination.

Coronal sections were then prepared from 3- to 4-month-old mice following EEG recordings. Phosphorylated S6 immunostaining revealed that tamoxifen injections effectively increased mTORC1 activity in tdTomato^+^ neurons electroporated with cRheb^CA^ compared with control neurons in littermate mice (Fig. 5b,c,e–g). In addition, cRheb^CA^-expressing neurons were cytomegalic as identified by a significant increase in their soma size compared with control neurons (Fig. 5b–d). As anticipated, late Rheb^CA^ expression did not lead to neuronal misplacement or the formation of white matter heterotopia (Fig. 5c). However, five of six cRheb^CA^-expressing mice displayed recurrent, Racine 3/5 seizures similar to mice expressing Rheb^CA^ (Fig. 5h,i), while no cGFP-expressing littermates developed seizures (*N*=6, *P*<0.01, two-proportioned *z*-test). These data strongly suggest that neuronal misplacement and white matter heterotopia are not required for seizure generation and that defects in neuronal connectivity and intrinsic electrogenic properties may be sufficient to induce spontaneous, convulsive seizures.

## Discussion

In this study, we report the development and full characterization of a model of type II FCD associated with spontaneous, convulsive seizures. In addition, using this experimental FCD model, we examined whether postpartum rapamycin treatment would be sufficient to prevent dysplasia and epilepsy and whether heterotopia of the cortex was a requirement for seizure generation.

In previous studies, increased mTORC1 activity was generated by means of *in utero* electroporation using one of three vectors: a Cre plasmid in conditional *Tsc1* mice, a constitutively active mTOR plasmid or a short hairpin RNA against *Tsc2.* Each led to neuronal misplacement, increased neuronal size and the presence of neurons with dysmorphic dendritic trees^22^,^23^,^24^. One of the studies also reported the presence of ectopic neurons in the white matter^22^. However, none of the lesions was associated with spontaneous, convulsive seizures, although one study reported decreased seizure threshold^22^. In all three studies, the electroporated regions were in the SSC, which may be inherently non-epileptogenic. Similarly, electroporation of Rheb^CA^ in the SSC in this study did not result in spontaneous, tonic–clonic seizures. We thus reasoned that generating dysplasia in an epileptogenic region would increase our chance of generating spontaneous seizures. Using a specific placement of the electrodes for current injection during electroporation, we successfully expressed plasmids in neurons destined for layer 2/3 of the mPFC. Changes of electrode placement during current injection allowed us to electroporate several regions of the mPFC, including the pre-orbital cortex, infralimbic cortex and ACC. Generating lesions in the mPFC led to spontaneous, convulsive seizures occurring at a frequency of 4–5 per day in 75% of the electroporated animals. We noted some variability in the number of seizures exhibited in the experimental animals during the 3-day EEG recordings. This variability could be accounted for by several parameters: the extent of the electroporated region, electroporation of the M1 region of the motor cortex and some variability in the age of the embryos at the time of electroporation. Correlation between seizure frequency and these parameters was not performed in this study, due to our focus characterizing the newly developed models of malformations and addressing some of the mechanisms involved in seizure generation.

On the basis of the cellular characterization performed in this study, the focal lesions were identified as type IIa FCDs. Indeed, we report the presence of dyslamination due to neuronal misplacement and white matter heterotopia (type I FCD), with the presence of cytomegalic, dysmorphic neurons, but no Balloons cells, which have a mixed neuro-glial phenotype. Rheb^CA^-expressing neurons somatically accumulate light chain neurofilament that are normally expressed at very low levels in somas of control neurons. The presence of enlarged neurons led to a significant increase in cortical thickness observed with anatomical MRI, similar to findings in patients with FCDs^39^. In addition, in animals experiencing seizures, gliosis was present throughout the entire cortex suggesting that epilepsy triggered gliosis in regions devoid of FCDs. In addition, gliosis was absent when the mice did not exhibit seizure activity, despite displaying FCDs in the SSC or mPFCs. These data support the idea that gliosis is not induced by the presence of FCDs alone without seizure activity and may not be the trigger of seizures. Nevertheless, whether gliosis contributes to the worsening of seizure severity (for example, stage or frequency) and additional damages (for example, blood–brain barrier disruption over time) needs to be addressed in further studies. Our model could provide novel insights into these issues by conducting EEG recordings from earlier time points to link GFAP expression to the onset and number of seizure episodes in comparisons or by performing longitudinal studies while manipulating astrocytic properties. Additional changes included alterations in connectivity, detected by increased dendritic complexity of affected neurons as well as DTI. It remains unclear whether changes in DTI reflect changes in dendritic or axonal connectivity. Nevertheless, layer 2/3 pyramidal neurons are callosal projection neurons and electroporated neurons project to the contralateral ACC and M1 cortex. The fact that most of the changes were in the ipsilateral cortex containing the FCDs suggests that increased dendritic complexity and perhaps increased cell size and overall disorganization of the cortex contribute to DTI alterations. Despite staining for a series of markers present in human balloon cells of type IIb FCDs, we did not find evidence of balloon cells in our FCD model. The lack of balloon cells, even in adult mice experiencing seizures for 10 months, suggests that additional events such as immune activation and inflammation could lead to engulfment of different cell types but not full degradation. Collectively, these features define our experimental FCD model as a typical type IIa FCD. In addition to serving as a model for type IIa FCDs, we propose that our model may also be useful for studying type IIb FCDs and TSC-associated cortical tubers because the only differences with our model are the absence of Balloon cells in FCDs and Giant cells in TSC, which are electrically silent and may not play a role in epileptogenesis.

In recent years, rapamycin has become the drug of choice for treating TSC-associated symptoms, primarily subependymal giant cell astrocytoma in an open label study^49^ and more recently seizures in children younger than 3 years old^50^,^51^,^52^, although one case study reported worsening of the seizure^53^. In animal models of TSC-associated malformations, prenatal and postnatal rapamycin treatments showed significant improvements in preventing premature death, decreased brain megalencephaly, limiting seizure activity, as well as gliosis, but did not fully prevented abnormal lamination or cytomegaly depending on the treatment regimens^13^,^43^,^44^,^45^,^46^,^47^,^48^. Studies have used different treatment regimens in different transgenic mouse models, thus limiting direct comparisons between studies. Here we show that rapamycin treatment starting at P1 fully rescued all cellular abnormalities, and prevented the generation of FCDs and seizures. However, as previously mentioned in other studies, treated mice showed significant developmental delays, including poor weight gain and delayed eye opening^44^. The developmental delays raise concerns and potential limitations of rapamycin for clinical use in young patients. Ultimately, it will be important to examine whether later onset of treatment with rapamycin (that is, once seizures have been diagnosed) will be sufficient to block seizure activity. Our model is thus amenable to assessing and quantifying the efficiency of different rapamycin treatment regimens in terms of timing and dosage on rescue of the cellular defects and seizure activity.

A previous study reported that seizures reinitiate after stopping rapamycin treatments^48^. The prior study used transgenic mice that showed massive forebrain enlargement, including increased mTORC1 activity in all cortical pyramidal neurons and many cortical astrocytes; the extent of cortical malformation in this alternative model preclude assessing whether transient rapamycin treatment was sufficient to prevent seizures that occurred from a local malformation. In the present study, we withdrew rapamycin in a cohort of mice and found that, despite rescuing neuronal misplacement and preventing heterotopia, seizures reoccurred. We examined seizure activity at 2- to 3-month post-rapamycin withdrawal, but it will be important in future studies to perform a longitudinal EEG study to assess the length of time, in which animals remain seizure free post rapamycin withdrawal. These data also suggest that neuronal misplacement and heterotopia are not necessary for seizure activity. This latter conclusion is further supported using inducible Rheb^CA^ showing that spontaneous, convulsive seizures occurred in mice when layer 2/3 pyramidal neurons had enhanced mTORC1 activity and cytomegaly, but were properly positioned. Clinically, this is an unfortunate finding because it implies that prolonged rapamycin treatment will be needed in patients with FCDs to decrease mTORC1 activity and prevent neuronal hypertrophy.

The mechanisms of seizure generation remain unclear in FCDs or cortical tubers in TSC, but can be further evaluated in our model. The electrophysiological properties of the neurons can be readily assessed using electrophysiological recordings in acute slices or *in vivo*. Similarly, silencing the cytomegalic cells using genetically encoded silencer (for example, halorhodopsin or engineered G-protein coupled DREADD receptor) would address whether altering the biophysical properties of the cells, without restoring their proper morphology and thus connectivity, would rescue seizure activity. A long standing question is whether seizure activity starts in the malformation containing dysmorphic neurons or in the surrounding region that may display abnormal connectivity. However, due to a limited spatial resolution of our EEG recording system, this question could not be addressed. Using a more focal EEG probe or an electrode grid with high spatial resolution would be required to address this important mechanistic question. Furthermore, if FCD neurons are found to initiate seizures, this should be confirmed with the use of genetically encoded silencer.

In conclusion, our model of FCDs will lead to further experimental studies to address fundamental questions regarding the mechanisms of epileptogenesis and epileptic activity in FCDs and help to identify and differentiate causal alterations from epiphenomenon associated with seizures. In addition, considering that there was no decrease in animal survival in our new model, it provides a powerful new approach for examining the outcome of seizure activity on cognitive and psychiatric behaviours and for longitudinal analyses of the effects of drug treatments on anatomy and behaviour.

## Methods

### Animals

Research protocols were approved by the Yale University Institutional Animal Care and Use Committee. All experiments were performed on CD-1 (Charles River), an outbred strain of mice of either gender.

### *In utero* electroporation and plasmids

Each DNA plasmid was diluted in sterile phosphate-buffered saline (PBS, pH 7.4) to a final concentration of 1.5–3 μg μl^−1^ (specific concentrations below). About 1 μl of DNA solution, containing 0.1% fast green added as injection tracer, was injected into the lateral ventricle of E15.5±0.5 fetuses with a glass pipette. After injecting all fetuses in a single uterine horn with manual pressure, PBS-soaked tweezer-type electrodes (model 520, BTX) were positioned on heads of the fetuses across the uterine wall and six square pulses at 42 V, 50 ms duration, 950 ms intervals were applied using a pulse generator (ECM830, BTX).

To generate the epilepsy-associated FCD, a DNA solution composed of, pCAG-Rheb^S16H^ (Rheb^CA^, 1.5 μg μl^−1^) and pCAG tdTomato (tdTomato^CAG^, 1.5 μg μl^−1^, Addgene) was injected in a half of all fetuses, while pCAG-GFP (GFP^CAG^, 3 μg μl^−1^, Addgene) containing solution was injected in the remaining littermates as controls. To generate epilepsy-associated FCD without heterotopia and dyslamination, a solution of loxP-containing vector pCALNL-Rheb^S16H^ (cRheb^CA^, 1.5 μg μl^−1^)+a Tamoxifen-inducible pCAG-ER^T2^CreER^T2^ (3 μg μl^−1^, Addgene)+pCAG-GFP (1.5 μg μl^−1^) was injected in half of the fetuses while the remaining half received a solution containing pCALNL-GFP (cGFP, 1.5 μg μl^−1^, Addgene)+pCAG-ER^T2^CreER^T2^ (3 μg/μl)+pCAG tdTomato (1.5 μg μl^−1^) as controls.

Mice were prescreened for successful electroporation via expression of fluorescent protein markers on fluorescence-enabled stereomicroscope (SZX16, Olympus) before recruitment for EEG and behavioural seizure monitoring.

### Seizure detection and analysis

Animals were randomly assigned an arbitrary identification number without knowing the experimental condition before implanting EEG and EMG electrodes for double-blind identification later. EEG/EMG headmounts (Pinnacle Technology, Inc.) were attached with four stainless steel machine screws (000–120; 0.1 in anterior and 0.125 in. posterior)^54^ to the skulls of successfully electroporated animals of at least 2 months of age. After 1 week of recovery, EEG/EMG preamplifiers (Pinnacle Technology, Inc.) were attached to the implants and to an electrical commutator (Pinnacle Technology, Inc.) to allow tethered recording from freely moving animals. EEG and EMG (sampled at 400 Hz) were recorded with digital video continuously for 72 consecutive hours for each animal.

Epileptiform electrical activity was analysed *post hoc*, while blinded to the condition, using Sirenia Seizure Pro software (Pinnacle Technology, Inc.) to automatically and objectively identify possible seizure epochs. An automated line length search method was applied to all recorded EEG channels with the threshold set at 4,000 length per s using a 15 s search window with a 0.25 s sliding window. Identified episodes were verified manually with EEG and video inspection. Convulsions that reached Racine stage 3–5 (ref. 55), from forelimb clonus to rearing and falling with forelimb clonus, were counted as a seizure. Seizure duration was defined from the onset of convulsion to cessation of all motor movement. Spectral analyses were conducted on 10 s windows before, during and after verified seizure epochs using Pinnacle Seizure Pro software (Pinnacle Technology, Inc.).

### Brain slice preparation and Immunohistochemistry

Mice were deeply anaesthetised with pentobarbital (50 mg kg^−1^) and perfused transcardially with ice-cold PBS (pH 7.4) followed by ice-cold paraformaldehyde (PFA, 4%). Perfused brains were also drop fixed in 4% PFA for an additional hour after removal from the skull. Fixed brains were cryoprotected with 30% sucrose in PBS overnight at 4 °C and serially sectioned into 50 μm thick sections, using a freezing microtome. Sections were blocked for 1 h at room temperature in blocking buffer consisting of 2% BSA and 0.3% Triton X-100 in PBS. Floating sections were incubated overnight at 4 °C in primary antibodies diluted in blocking buffer. Following three washes in PBS and an additional 15 min in blocking solution at room temperature, sections were then incubated with Alexa Fluor-tagged secondary antibodies (Supplementary Table 1) at 1:1,000 dilution for 2 h at room temperature. ProLong Gold antifade reagent (Life Technologies) is used to mount and preserve stained sections. While blinded to the conditions of the experiment, fluorescence micrograms of serial brain sections were acquired under a fluorescence-enabled stereomicroscope (SZX16, Olympus) at × 0.8 magnification. Stained sections were acquired using a fluorescence confocal microscope (FV1000, Olympus) also in a blind manner. Z-stack images were acquired on the confocal microscope with a × 20 dry objective (N.A. 0.75, Olympus). Images were reconstructed using Imaris 4.0 (Bitplane AG) and Photoshop CS3.

Antibodies included anti-: GABA (Sigma, A2052, 1:4,000), GFAP (DAKO, Z0334, 1:4,000), myelin basic protein (Abcam, ab40390, 1:2,000), Nestin (Novus Bio., NM100-1604, 1:300), NeuN (Millipore, MAB377, 1:4,000), NF light chain (Cell Signaling, C28E10, 1:500), pS6 (S240/244, Cell Signaling, D68F8, 1:4,000), SMI 311 (Covance, SMI 311 R, 1:4,000) and Vimentin (MBL, JM-3634-100, 1:500). The host animal is listed in Supplementary Table 1.

All analyses were conducted with the analyser knowing only the arbitrarily assigned animal ID (independent of electroporation condition) from which images were taken. Fluorescence intensities and cell sizes were quantified using ImageJ 1.39t (Freeware, Wayne Rasband, NIH). GABA-immunoreactive cells were counted using ImageJ after converting fluorescence micrograms to 8 bit and automatically thresholded. Cells ipsilateral to the side of electroporation were automatically counted with the ‘Analyze Particle' function in ImageJ with the following parameters: minimum cell size 19.6 μm^2^, circularity 0.40–0.00.

### Analyses of neuron features and pS6 immunostaining

To quantify laminar positioning, all electroporated cells containing DAPI+ nuclei were manually counted. Cells within 300 μm from the midline were considered layer 2/3 cells and correctly located, while cells outside that boundary were considered misplaced. Total pS6 was quantified in ImageJ by measuring average pS6 intensities within a region of interest (ROI) that outlines the electroporated soma and multiplied by the surface area of their respective ROI. Somatic ROIs were subsequently used for cell size analysis.

### DTI and cortical thickness analysis

Twelve mice (six in each group) used in MRI and DTI studies were perfused, fixed and extracted as described previously^56^ and summarized as follows. The *ex vivo* brains were soaked in PBS (three times for 10 min in 10 ml PBS) and tapped dry to remove excess PFA before the MR scans. Before imaging, the brain was placed into a custom-built MR compatible syringe filled with Fluroinert- a proton-free MRI susceptibility matching fluid (Sigma Aldrich, MO). MRI and DTI scans were acquired on a 9.4 T horizontal bore magnet (Bruker) with a custom-made proton radio frequency volume coil. The DTI experiments were performed using the Stejskal-Tanner spin-echo diffusion-weighted sequence with a diffusion gradient of 5 ms and a delay between the two diffusion gradients of 15 ms. Twenty-four contiguous coronal slices of 0.5 mm thickness were obtained using a repetition time (TR) of 2 s and an echo time (TE) of 25.1 ms. Two 1 ms Shinnar-Le Roux (SLR) pulses were used for excitation and inversion. Twenty averages were acquired for each slice and the 128 × 64 pixel resolution images were zero-filled to 256 × 256 pixel resolution, resulting in an in-plane isotropic spatial resolution of 100 × 100 μm. Sixteen diffusion weighting directions with *b*=1,000 s mm^−2^ were used for the DTI acquisitions. DTI processing and analysis was performed blindly as previously described^56^,^57^. MRI T_1_-wegithed images were acquired with fast spin echo sequence (TR of 3 s and TE of 40 ms) using the same geometric parameters as in DTI scans. Cortical thickness of the T_1_-weighted images was measured for ipsilateral and contralateral cortex for both control and Rheb^CA^ mice using MATLAB (Version 2013a).

### Acute slice preparation and whole-cell recording

One-month-old mice were used for acute slices. Tissue was dissected and sliced in ice-cold artificial cerebral spinal fluid (aCSF) oxygenated with 95% O_2_/5%CO_2_. The aCSF contained (all in mM): 124 NaCl, 3 KCl, 1.25 NaH_2_PO_4_, 1 MgSO_4_, 26 NaHCO_3_, 10 Dextrose, 2 CaCl_2_, 0.4 ascorbate, 4 Na-Lactate, 2 Na-Pyruvate (290±5 mOsm kg^−1^, pH 7.2). Coronal sections (350 μm) were prepared using a vibratome (Vibratome 1000). Sections were incubated in aCSF at 32 °C for 45 min before returning to room temperature (25 °C) where they were kept for 8–10 h during experimentation. TdTomato-positive neurons in layer 2/3 of ACC were visualized using epifluorescence on an Olympus BX51WI microscope with a × 40 water immersion objective (Olympus, LUMPlanFL/IR). Whole-cell recordings were performed at 28 °C using pulled glass pipettes (4–7 ΩM) filled with internal solution (in mM: 130 KCl, 10 HEPES, 10 di-tris-phosphocreatine, 1 EGTA, 0.1 CaCl_2_, 1.5 MgCl_2_, 4 Na_2_-ATP, 0.4 Na-GTP). Internal solution also included 0.5% Neurobiotin (Vector Labs) for visualizing cell morphology. Recordings were acquired with an amplifier (Axopatch 200B, Molecular Devices). Neurobiotin fills were revealed with Streptavidin conjugated Alexa Fluor 633 (Thermo Fisher Scientific, formerly Life Technologies).

### Statistical analyses

Data were plotted in Prism 6 (GraphPad Software, Inc.). Statistical significance was determined using unpaired two-tailed Student's *t*-test and two-way analysis of variance with Sidak's multiple comparisons post-test with *P*<0.05 for significance for all experiments. Two-proportion z-test was used without Yates' correction to test the significance of epileptogenicity between control and experimental groups, with *P*<0.01 for significance. Data are presented as mean±s.e.m.

### Data availability

The authors declare that the data supporting the findings of this study are available within the article and its supplementary information files.

## Additional information

**How to cite this article:** Hsieh, L. S. *et al*. Convulsive seizures from experimental focal cortical dysplasia occur independently of cell misplacement. *Nat. Commun.* 7:11753 doi: 10.1038/ncomms11753 (2016).

## Supplementary Material

Supplementary Information Supplementary Figures 1 - 4 and Supplementary Table

Supplementary Video 1 A video of Racine stage 5 seizure in our model of FCD. A 50 second video (10 fps) of a Rheb^CA^ electroporated mouse having a representative spontaneous tonic clonic seizure.
