# Supplementary material for: Convulsive seizures from experimental focal cortical dysplasia occur independently of cell misplacement
Source: Nat Commun. 2016 Jun 1;7:11753. doi: 10.1038/ncomms11753 (PMC4895394; doi:10.1038/ncomms11753)
Supplement: Supplementary Information — Supplementary Figures 1 - 4 and Supplementary Table [file ncomms11753-s1.pdf]

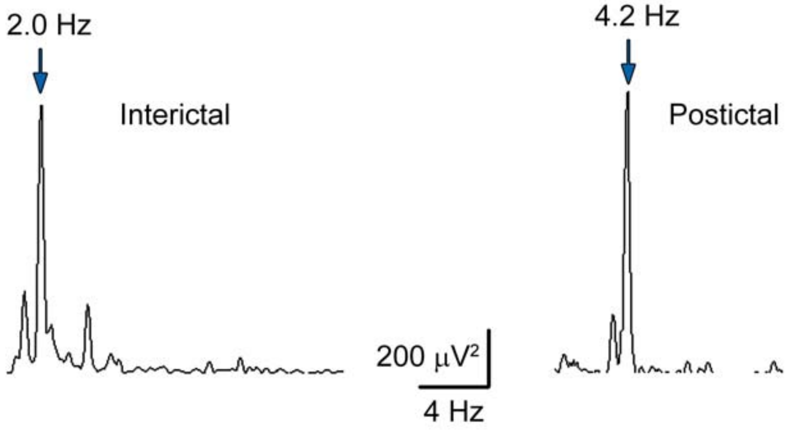

Supplementary Figure 1. Power spectra of the pre- and post-ictal periods for the EEG trace shown in Figure 1f.

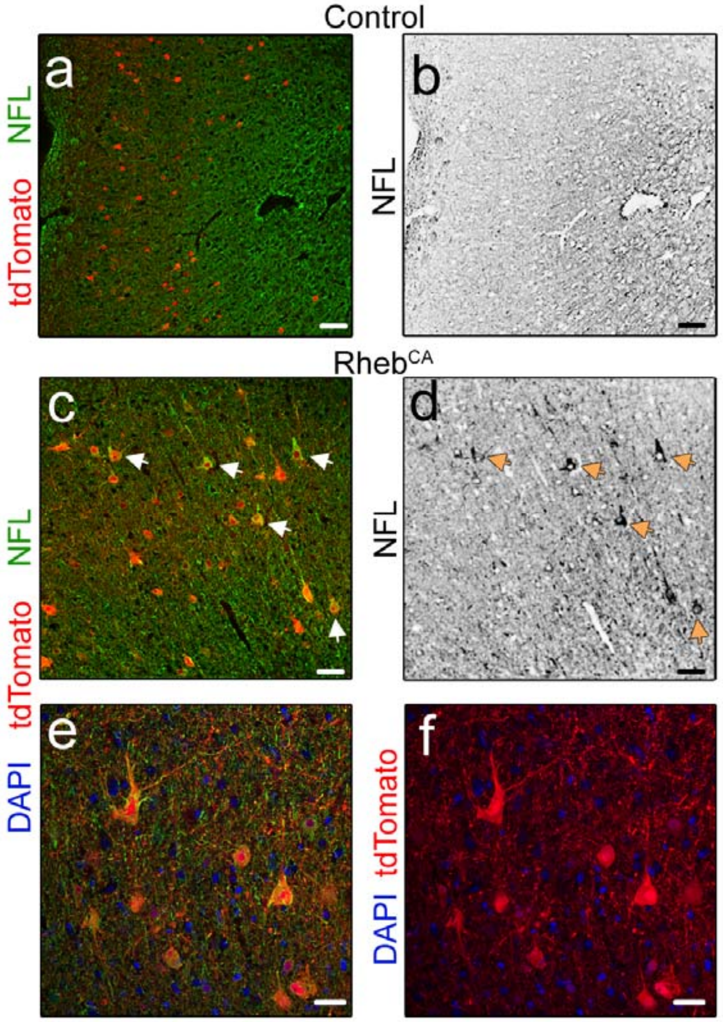

Supplementary Figure 2. Neurofilament light chain accumulates in the soma of dysmorphic neurons. (a and c) Immunostain of neurofilament light chain (NF-L, green) in a coronal section containing control (a) or Rheb^{CA} (c) electroporated neurons (in red). Rheb^{CA}-electroporated neurons (arrows) accumulate NF-L immunoreactivity in the soma. **(b and d)** Gray scale rendering of the image in (a and c) without showing electroporated cells. **(e and f)** Higher magnification of the neurons shown in (c) illustrating that NF-L-immunopositive neurons are dysmorphic. Scale bar: 50 μm.

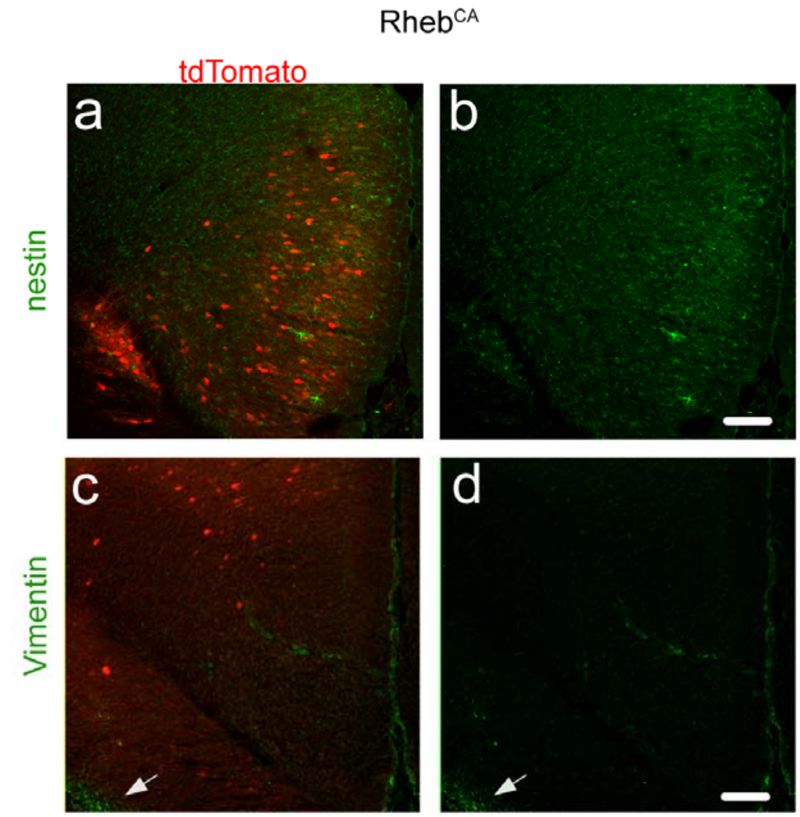

Supplementary Figure 3. Images of immunostaining for nestin (a and b) and vimentin (b and c) in coronal sections containing Rheb^{CA}-electroporated neurons in the mPFC of mice displaying seizures. The white arrow points towards the neurogenic zone illustrating positive staining with the vimentin antibody. Scale bar: 100 µm

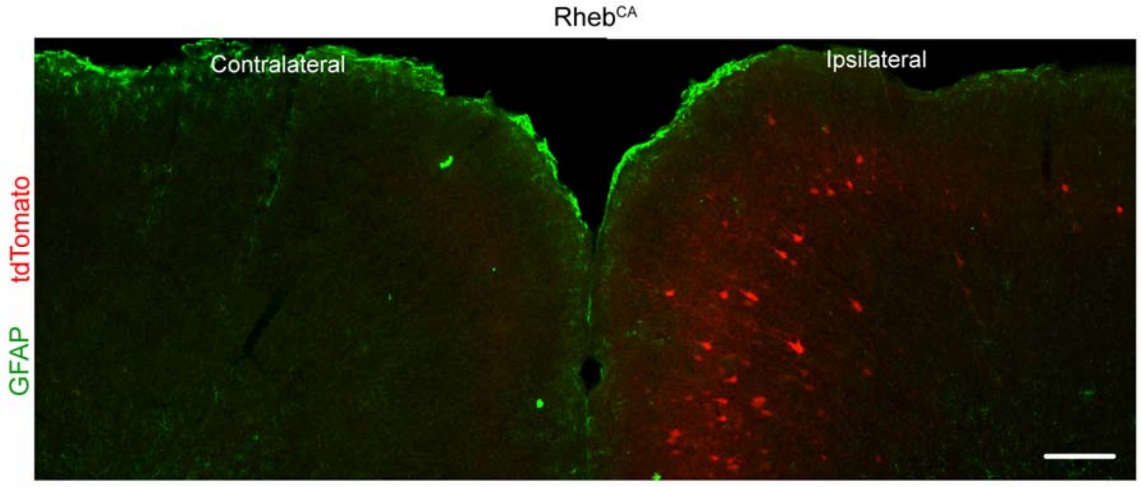

Supplementary Figure 4. GFAP immunostaining (green) in coronal sections containing Rheb^{CA}-electroporated neurons (red) in the anterior cingulate cortex of mice that did not exhibit seizures.

Supplementary Table 1: Primary and Secondary antibodies

Antibody	Company	Catalog Number	Host animal	Concentration used in IHC
Primary				
GABA	Sigma	A2052	Rabbit	1:4000
GFAP	DAKO	Z0334	Rabbit	1:4000
MBP	Abcam	ab40390	Rabbit	1:2000
Nestin	Novus Biologicals	NB100-1604	Chicken	1:300
NeuN	Millipore	MAB 377	Mouse	1:4000
NF-L	Cell Signaling	C28E10	Rabbit	1:500
pS6 (S240/244)	Cell Signaling	D68F8	Rabbit	1:4000
SMI 311	Covance	SMI-311R	Mouse	1:4000
Vimentin	MBL	JM-3634-100	Rabbit	1:500
DAPI	Life Technologies	D1306		1:36000
Secondary				
α Mouse IgG Alexa Fluor 633	Life Technologies	A-21052	Goat	1:1000
α Mouse IgG Alexa Fluor 488	Life Technologies	A-11001	Goat	1:1000
α Rabbit IgG Alexa Fluor 555	Life Technologies	A-31572	Donkey	1:1000
α Rabbit IgG Alexa Fluor 647	Life Technologies	A-31573	Donkey	1:1000
α Chicken IgY Alexa Fluor 488	Life Technologies	A-11039	Goat	1:1000
α Goat IgG Alexa Fluor 568	Life Technologies	A-11057	Donkey	1:1000
